# Effects of Post Ischemia-Reperfusion Treatment with Trimetazidine on Renal Injury in Rats: Insights on Delayed Renal Fibrosis Progression

**DOI:** 10.1155/2018/1072805

**Published:** 2018-07-02

**Authors:** Jin Ha Park, Ji Hae Jun, Jae-Kwang Shim, Eun Jung Shin, Eunah Shin, Young-Lan Kwak

**Affiliations:** ^1^Department of Anesthesiology and Pain Medicine, Yonsei University College of Medicine, Seoul, Republic of Korea; ^2^Anesthesia and Pain Research Institute, Yonsei University College of Medicine, Seoul, Republic of Korea; ^3^Yonsei Cardiovascular Research Institute, Yonsei University College of Medicine, Seoul, Republic of Korea; ^4^Department of Pathology, CHA Gangnam Medical Center, CHA University, Seoul, Republic of Korea

## Abstract

Even after recovery from acute kidney injury, glomeruli remain vulnerable to further injury by way of interstitial fibrosis. This study is aimed at elucidating the effects of post ischemia-reperfusion (I/R) treatment with trimetazidine on the progression to renal fibrosis as well as short- and intermediate-term aspects. Trimetazidine 3 mg/kg or 0.9% saline was given intraperitoneally once upon reperfusion or daily thereafter for 5 d or 8 w. Renal histologic changes and related signaling proteins were assessed. After 24 h, post I/R treatment with trimetazidine significantly reduced serum blood urea nitrogen and creatinine levels and tubular injury accompanied with upregulation of hypoxia-inducible factor- (HIF-) 1*α*, vascular endothelial growth factor (VEGF), and Bcl-2 expression. After 5 d, post I/R treatment with trimetazidine reduced renal tubular cell necrosis and apoptosis with upregulation of HIF-1*α*-VEGF and tissue inhibitors of metalloproteinase activities, attenuation of matrix metalloproteinase activities, and alteration of the ratio of Bax to Bcl-2 levels. After 8 w, however, post I/R treatment with trimetazidine did not modify the progression of renal fibrosis. In conclusion, post I/R treatment with trimetazidine allows ischemic kidneys to regain renal function and structure more rapidly compared to nontreated kidneys, but not enough to resolute renal fibrosis in long-term aspect.

## 1. Introduction

Acute kidney injury (AKI) is one of the most frequent complication in hospitalized patients, which is believed to be the consequence of ischemia-reperfusion (I/R) injury [1–3]. Of importance, glomeruli remain vulnerable to additional injury after AKI and foster progression to chronic kidney disease (CKD) by way of interstitial fibrosis, regardless of the degree of recovery from AKI [4]. This is supported by clinical evidence that even a transient increase in serum creatinine has consistently been recognized as a portent of adverse outcome [5].

As an innate response to renal I/R injury and AKI, activation of hypoxia-inducible factor- (HIF-) 1*α* stimulates various cell survival signaling pathways, including a principal pathway that attenuates oxidative stress via expression of vascular endothelial growth factor (VEGF) [6, 7]. Signals essential for renal protection and repair immediately after I/R injury, however, could result in delayed renal fibrosis and CKD [8–10]. In that context, controlling excessive cell proliferation and extracellular matrix production would be of key issue to establish therapeutic options that can arrest the progression to renal fibrosis.

Trimetazidine (TMZ, 1-[2,3,4-trimethoxybenzyl]piperazine dihydrochloride), an anti-ischemic agent in current use, has been shown to reduce myocardial and renal I/R injury at both the cellular and mitochondrial levels [11, 12]. Putative mechanisms involved reduced intracellular acidosis, preserved ATP stores, and inhibited inflammatory reactions [13, 14]. More importantly, pretreatment with TMZ attenuated the progression of renal I/R injury by mitigating apoptosis and interstitial fibrosis in association with promoted release of HIF-1*α* [15, 16]. Yet, the majority of studies have focused on the early effects of TMZ pretreated before I/R injury [11, 14, 17]. However, the development of AKI is difficult to predict and also challenged by the inability of timely diagnosis, which render pretreatment measures not clinically feasible.

Thus, this study is aimed at elucidating the effects of post I/R treatment with TMZ on the progression to renal fibrosis as well as short- and intermediate-term aspects and at investigating relevant signaling pathways.

## 2. Materials and Methods

### 2.1. Animals

All experiments were approved by the committee for the Care and Use of Laboratory Animals, Yonsei University College of Medicine, and were performed conforming to the *Guide for the Care and Use of Laboratory Animals* published by the US National Institutes of Health [18].

Male Sprague-Dawley rats (10–12 w old, 250–300 g) were anesthetized with Rompun (vial Korea, 10 mg/kg, intraperitoneally (ip)) plus Zoletil 50 (Virbac Korea, 30 mg/kg, ip). The rats were intubated with a 16-gauge catheter and artificially ventilated (Harvard Apparatus 683, Holliston, MA) at 30–35 cycles/min. The body temperature was continuously monitored throughout the experiment and maintained around 37°C using a heating pad.

### 2.2. Experimental Models and Study Groups

We employed an established rodent model of renal I/R injury having the longest period of ischemia to induce an appropriate degree of renal injury while allowing long-term survival [19, 20]. After midline incision to expose both kidneys, the left kidney was removed in all rats except those in the sham group. The right renal pedicle was clamped for 45 min with an atraumatic microvascular clamp and then reperfused for 24 h, 5 d, or 8 w to perform short-, intermediate-, and long-term assessment, respectively.

The animals were randomly assigned to four groups: (1) sham (*n* = 10), 0.9% saline only; (2) I/R control (IRC) (*n* = 15), I/R + 0.9% saline; (3) single TMZ posttreatment after I/R (TMZ^S^) (*n* = 15), I/R + TMZ single administration upon reperfusion; and (4) daily TMZ posttreatment after I/R (TMZ^D^) (*n* = 15), I/R + daily TMZ administration for 5 d or 8 w starting upon reperfusion.

To examine the effect of TMZ on renoprotection, TMZ (3 mg/kg, Sigma, St. Louis, MO) was administered to the TMZ-treated groups while the sham and IRC groups received equivalent amounts of 0.9% saline via ip. The dose of TMZ was chosen based on our pilot study that investigated the optimal dose of TMZ in terms of attenuating apoptotic cell death after renal I/R injury (3 mg/kg versus 1 and 5 mg/kg, data not shown).

### 2.3. Blood Urea Nitrogen (BUN) and Creatinine Analysis

Serum samples were obtained at 24 h, 5 d, and 8 w after reperfusion and assayed for BUN and creatinine levels using the picric acid and diacetyl monoxime methods [21], respectively.

### 2.4. TUNEL Assay

Detection of apoptosis on paraffin sections from each group was examined using the terminal deoxynucleotidyl transferase-mediated uridine triphosphate nick end labeling (TUNEL) [22]. Five visual fields from each sample block were randomly selected and analyzed by a blind observer using a microscope (×400). The apoptotic index was analyzed (apoptotic cells/total cells × 100%) from a total of 20 fields per sample.

### 2.5. Renal Histopathology Examination

Paraffin-embedded kidney tissues were cross sectioned (5-6 specimens per group) through the midpoint to measure histologic damage. Periodic acid-Schiff (PAS) staining was performed and the degree of tubular damage was graded on a scale from 1 to 4. Grading and definitions for cell degradation, necrosis, and neutrophil infiltration are listed in Table 1 [8]. Masson's trichrome staining was used to assess renal interstitial fibrosis [23]. Percentages of positively stained blue areas on Masson's trichrome-stained sections were assessed by microscopic review by a pathologist (Eunah Shin) to estimate the level of fibrosis. Vascular and perivascular areas were excluded from the estimation.

### 2.6. Immunoblot Analysis

After processing, equal amounts of protein from each group underwent immunoblot assay as described previously [24]. Proteins were separated on sodium dodecyl sulfate-polyacrylamide gel electrophoresis and immunoblotted with anti-VEGF (Santa Cruz, CA), anti-matrix metalloproteinase- (MMP-) 2, anti-MMP-9, anti-tissue inhibitor of metalloproteinase- (TIMP-) 1, anti-TIMP-2 (Calbiochem, USA), anti-Bcl-2, anti-Bax, anti-HIF-1*α*, and anti-actin (all from Cell Signaling Technology, Beverly, MA). Each analysis was performed at least three times.

### 2.7. Statistical Analysis

All data were expressed as mean ± standard deviation. Statistical analysis was performed using one-way analysis of variance (ANOVA) or Student's *t*-test followed by Bonferroni correction. Values of *P* < 0.05 were considered statistically significant.

## 3. Results

### 3.1. Post I/R Treatment with TMZ Reduced Short-Term Renal Dysfunction

**Figure 1:**
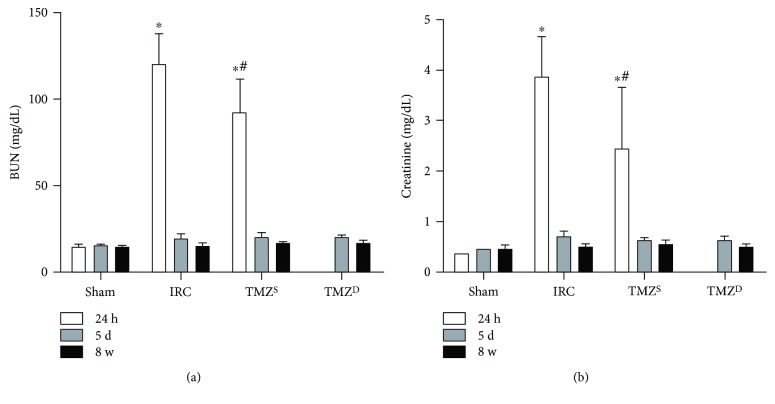
Effects of postischemia-reperfusion (I/R) treatment with TMZ on renal function at different time points. Renal I/R injury produced a significant increase in serum BUN (a) and creatinine levels (b), which were significantly attenuated in the TMZ^S^ group at 24 h after reperfusion. The elevated serum BUN and creatinine levels returned to their respective baseline values in all groups at 5 d after renal I/R injury and were at similar levels at 8 w after renal I/R injury without any intergroup differences. TMZ = trimetazidine; BUN = blood urea nitrogen. Sham = rats not underwent I/R; IRC = rats underwent ischemia (45 min)-reperfusion (24 h, 5 d, and 8 w); TMZ^S^ = rats treated with TMZ (3 mg/kg) upon reperfusion; TMZ^D^ = rats treated with TMZ (3 mg/kg) once daily for 5 d or 8 w starting upon reperfusion. ^∗^*P* < 0.05 versus sham, ^#^*P* < 0.05 versus IRC.

Renal I/R injury produced a significant increase in serum BUN and creatinine levels, which were significantly attenuated in the TMZ^S^ group at 24 h after reperfusion. The elevated serum BUN and creatinine levels returned to their respective baseline values in all groups at 5 d after renal I/R injury and were at similar levels at 8 w after renal I/R injury without any intergroup differences (Figures 1(a) and 1(b)).

### 3.2. Post I/R Treatment with TMZ Mitigated Renal Tubular Apoptotic Cell Death and Cell Necrosis at 24 h and 5 d but Not at 8 w after I/R Injury

**Figure 2:**
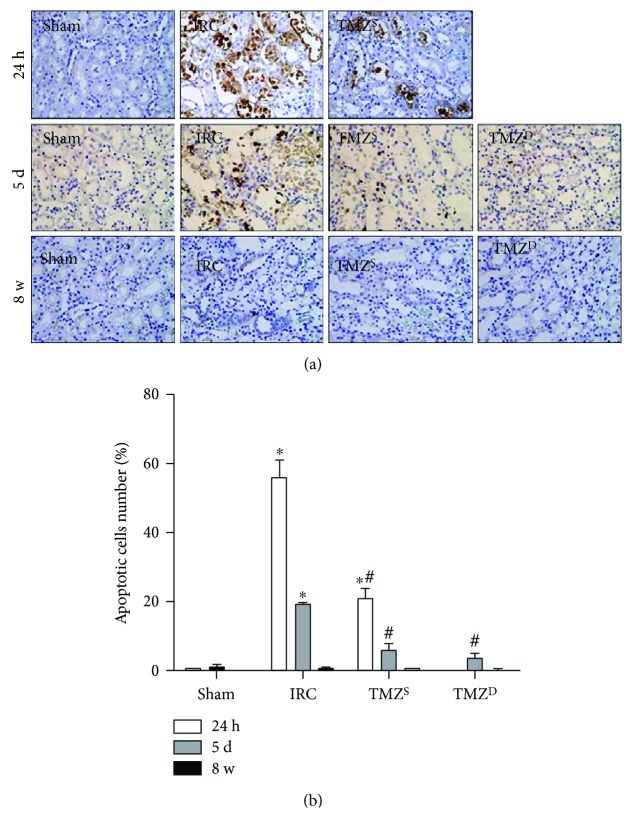
Effects of postischemia-reperfusion (I/R) treatment with TMZ on renal tubular apoptotic cell death. Representative histology of renal tubules after TUNEL assay (a) and a percentage of apoptotic cells (apoptotic cells/total cells) (b). Compared to the sham groups, I/R injury resulted in significant apoptotic cell death in the IRC groups at 24 h and 5 d after I/R injury. TMZ significantly attenuated apoptotic cell death compared to the IRC groups at 24 h and 5 d after I/R injury. At 8 w after I/R injury, TUNEL assay of renal tissues demonstrated almost full recovery of histopathologic findings in the IRC and the TMZ-treated groups, without intergroup differences. TMZ = trimetazidine; TUNEL = terminal deoxynucleotidyl transferase-mediated uridine triphosphate nick end labeling. Sham = rats not underwent I/R; IRC = rats underwent ischemia (45 min)-reperfusion (24 h, 5 d, and 8 w); TMZ^S^ = rats treated with TMZ (3 mg/kg) upon reperfusion; TMZ^D^ = rats treated with TMZ (3 mg/kg) once daily for 5 d or 8 w starting upon reperfusion. ^∗^*P* < 0.05 versus sham, ^#^*P* < 0.05 versus IRC.

**Figure 3:**
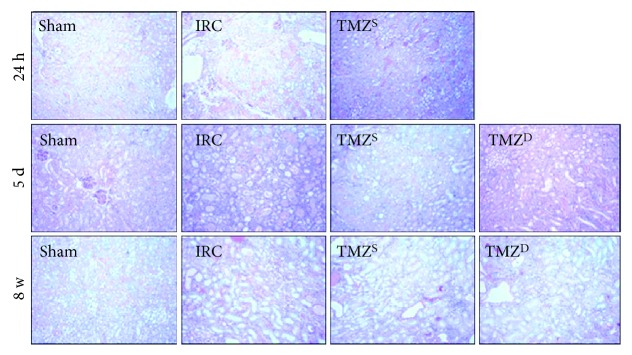
Effects of postischemia-reperfusion (I/R) treatment with TMZ on renal tubular cell necrosis. Representative histology of renal tubules after and PAS staining. Compared to the sham groups, I/R injury resulted in significant necrosis in the IRC groups at 24 h and 5 d after I/R injury. TMZ significantly attenuated cell necrosis compared to the IRC groups at 24 h and 5 d after I/R injury. At 8 w after I/R injury, PAS staining of renal tissues demonstrated almost full recovery of histopathologic findings in the IRC and the TMZ-treated groups, without intergroup differences. TMZ = trimetazidine; PAS = periodic acid-Schiff. Sham = rats not underwent I/R; IRC = rats underwent ischemia (45 min)-reperfusion (24 h, 5 d, and 8 w); TMZ^S^ = rats treated with TMZ (3 mg/kg) upon reperfusion; TMZ^D^ = rats treated with TMZ (3 mg/kg) once daily for 5 d or 8 w starting upon reperfusion.

Compared to the sham groups, I/R injury resulted in significant apoptotic cell death (Figures 2(a) and 2(b)) and necrosis (Figure 3, Table 2) in the IRC groups at 24 h and 5 d after I/R injury. TMZ significantly attenuated apoptotic cell death and necrosis compared to the IRC groups at 24 h and 5 d after I/R injury (Figures 2 and 3, Table 2). At 8 w after I/R injury, TUNEL assay and PAS staining of renal tissues demonstrated almost full recovery of histopathologic findings in the IRC and the TMZ-treated groups, without intergroup differences (Figures 2 and 3).

### 3.3. Post I/R Treatment with TMZ Attenuated the Degree of I/R-Induced Changes in Bcl-2 and Bax Levels at 24 h and 5 d, but Not at 8 w after I/R Injury

**Figure 4:**
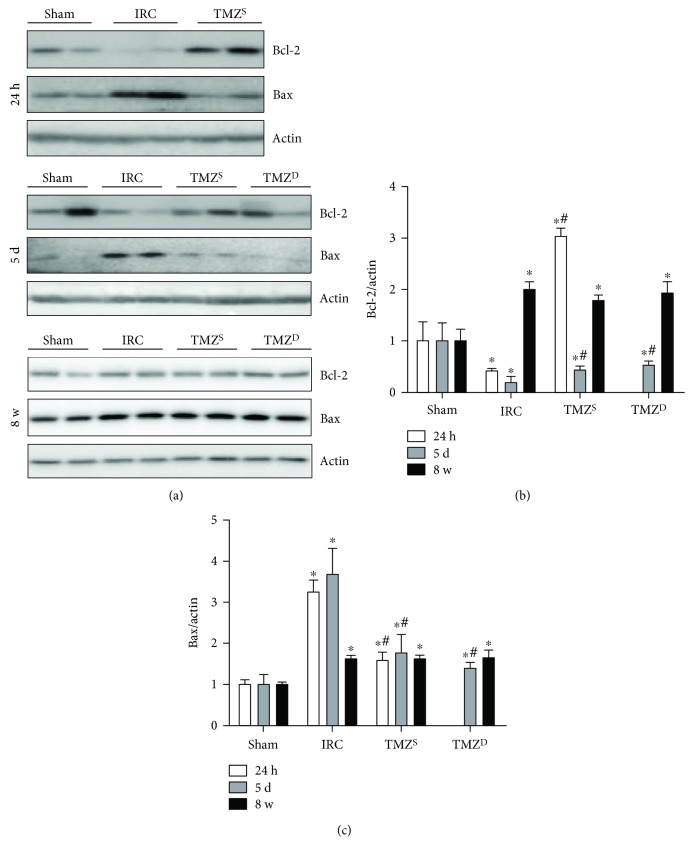
Effects of postischemia-reperfusion (I/R) treatment with TMZ on the expressions of Bcl-2 and Bax. Western blot analysis (a) and densitometric analysis (b, c) of the Western blot for Bcl-2 and Bax at 24 h, 5 d, and 8 w after I/R injury, respectively. Bcl-2 level was significantly decreased, and Bax level was significantly increased in the IRC groups compared to that in the sham groups at 24 h and 5 d after I/R injury. Bcl-2 level was significantly higher, and Bax level was significantly lower in the TMZ-treated groups than that in the IRC groups. At 8 w after renal I/R injury, Bcl-2 and Bax levels were significantly greater in the IRC and the TMZ-treated groups than those in the sham groups, without any intergroup differences between the IRC and the TMZ-treated groups. Sham = rats not underwent I/R; IRC = rats underwent ischemia (45 min)-reperfusion (24 h, 5 d, and 8 w); TMZ^S^ = rats treated with TMZ (3 mg/kg) upon reperfusion; TMZ^D^ = rats treated with TMZ (3 mg/kg) once daily for 5 d or 8 w starting upon reperfusion. ^∗^*P* < 0.05 versus sham, ^#^*P* < 0.05 versus IRC.

Bcl-2 level was significantly decreased, and Bax level was significantly increased in the IRC groups compared to that in the sham groups at 24 h and 5 d after I/R injury (Figures 4(a)–4(c)). In line with the antiapoptotic effect of TMZ, Bcl-2 level was significantly higher and Bax level was significantly lower in the TMZ-treated groups than that in the IRC groups (Figures 4(a)–4(c)). At 8 w after renal I/R injury, Bcl-2 and Bax levels were significantly greater in the IRC and the TMZ-treated groups than those in the sham groups, without any intergroup differences between the IRC and the TMZ-treated groups (Figures 4(a)–4(c)).

### 3.4. Post I/R Treatment with TMZ Enhanced the Expression of HIF-1*α* and VEGF at 24 h and 5 d, but Not at 8 w after I/R Injury

**Figure 5:**
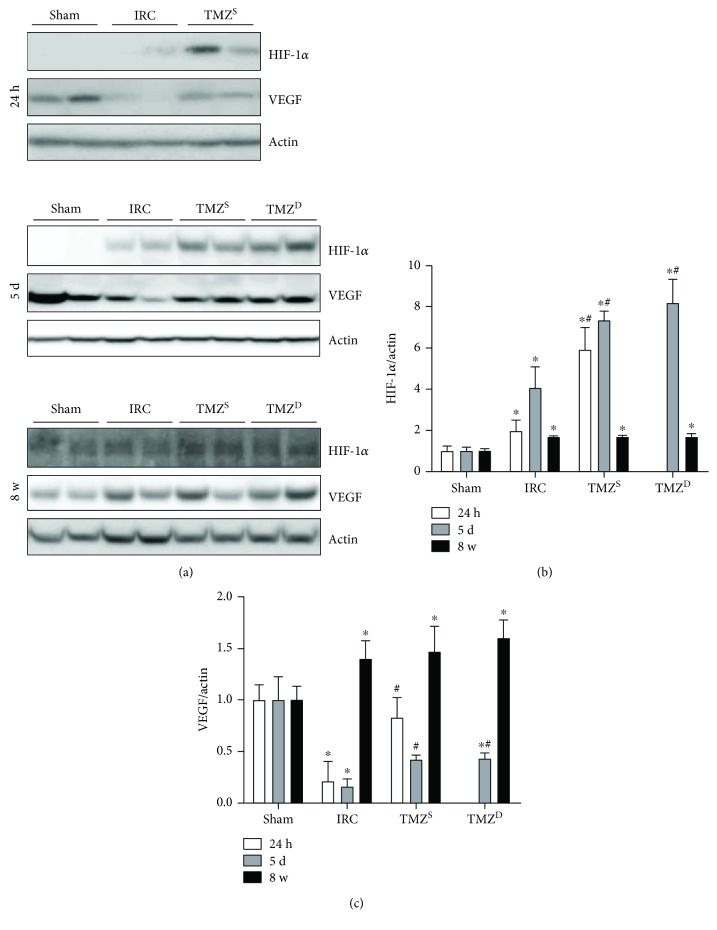
Effects of postischemia-reperfusion (I/R) treatment with TMZ on the expressions of HIF-1*α* and VEGF. Western blot analysis (a) and densitometric analysis (b, c) of the Western blot for HIF-1*α* and VEGF at 24 h, 5 d, and 8 w after I/R injury, respectively. Renal I/R injury induced increases in HIF-1*α* levels and decreases in VEGF levels in the IRC groups compared to that in the sham groups at 24 h and 5 d after I/R injury. Post I/R treatment with TMZ significantly increased both levels of HIF-1*α* and VEGF compared to the IRC groups at 24 h and 5 d after I/R injury. HIF-1*α* could not be detected in any groups at 8 w after I/R injury. At 8 w after I/R injury, VEGF levels were all significantly higher in the IRC and TMZ-treated groups compared to that in the sham groups without any intergroup differences between the IRC and the TMZ-treated groups. Sham = rats not underwent I/R; IRC = rats underwent ischemia (45 min)-reperfusion (24 h, 5 d, and 8 w); TMZ^S^ = rats treated with TMZ (3 mg/kg) upon reperfusion; TMZ^D^ = rats treated with TMZ (3 mg/kg) once daily for 5 d or 8 w starting upon reperfusion. ^∗^*P* < 0.05 versus sham, ^#^*P* < 0.05 versus IRC.

Renal I/R injury induced increases in HIF-1*α* levels (Figures 5(a) and 5(b)) and decreases in VEGF levels (Figures 5(a) and 5(c)) in the IRC groups compared to that in the sham groups at 24 h and 5 d after I/R injury. Post I/R treatment with TMZ significantly increased both levels of HIF-1*α* and VEGF compared to the IRC groups at 24 h and 5 d after I/R injury. HIF-1*α* could not be detected in any groups at 8 w after I/R injury (Figures 5(a) and 5(b)). At 8 w after I/R injury, VEGF levels were all significantly higher in the IRC and TMZ-treated groups compared to that in the sham groups without any intergroup differences between the IRC and the TMZ-treated groups (Figures 5(a) and 5(c)).

### 3.5. Post I/R Treatment with TMZ Did Not Attenuate Renal Fibrotic Change at 8 w after Renal I/R Injury

**Figure 6:**
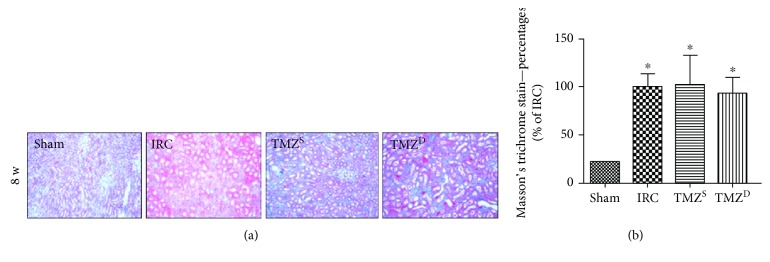
Effect of postischemia-reperfusion (I/R) treatment with TMZ on renal tubulointerstitial fibrosis. Representative histology (a) of renal tubules after Masson's trichrome staining and quantitative analysis (b) of tubulointerstitial fibrosis. Renal fibrosis was significantly more prominent in the IRC and TMZ-treated groups compared to that in the sham group at 8 w after renal I/R injury, without any intergroup differences between the IRC and TMZ-treated groups. TMZ = trimetazidine. Sham = rats not underwent I/R; IRC = rats underwent ischemia (45 min)-reperfusion (8 w); TMZ^S^ = rats treated with TMZ (3 mg/kg) upon reperfusion; TMZ^D^ = rats treated with TMZ (3 mg/kg) once daily for 8 w starting upon reperfusion. ^∗^*P* < 0.05 versus sham.

In Masson's trichrome staining, renal fibrosis was significantly more prominent in the IRC and TMZ-treated groups compared to that in the sham group at 8 w after renal I/R injury, without any intergroup differences between the IRC and TMZ-treated groups (Figures 6(a) and 6(b)).

**Figure 7:**
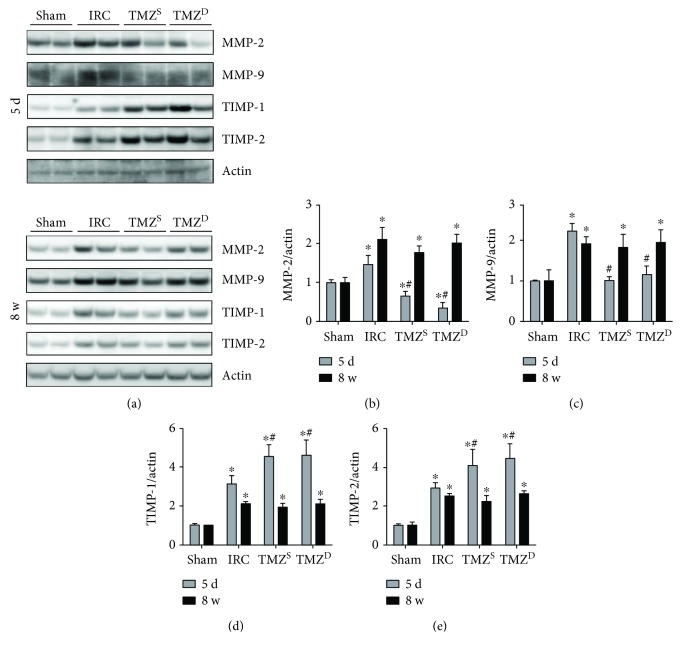
Effects of postischemia-reperfusion (I/R) treatment with TMZ on the expressions of MMPs and TIMPs. Western blot analysis (a) and densitometric analysis (b–e) of the Western blot for MMP-2, -9 and TIMP-1, -2 at 5 d, and 8 w after I/R injury, respectively. Post I/R treatment with TMZ reduced the degree of I/R-induced increases in MMP-2 and MMP-9 levels and further increased the levels of TIMP-1 and TIMP-2 at 5 d after renal I/R injury compared to the IRC group. At 8 w after I/R injury, MMP levels in the TMZ-treated groups were similar to those of the IRC groups, which were all significantly greater than those of the sham groups. TIMP levels in the TMZ-treated groups became also comparable to those of the IRC groups, which were significantly greater than those of the sham groups. TMZ = trimetazidine. Sham = rats not underwent I/R; IRC = rats underwent ischemia (45 min)-reperfusion (5 d or 8 w); TMZ^S^ = rats treated with TMZ (3 mg/kg) upon reperfusion; TMZ^D^ = rats treated with TMZ (3 mg/kg) once daily for 5 d or 8 w starting upon reperfusion. ^∗^*P* < 0.05 versus sham, ^#^*P* < 0.05 versus IRC.

Post I/R treatment with TMZ reduced the degree of I/R-induced increases in MMP-2 and MMP-9 levels (Figures 7(a)–7(c)) and further increased the levels of TIMP-1 and TIMP-2 (Figures 7(a), 7(d), and 7(e)) at 5 d after renal I/R injury compared to the IRC group. At 8 w after I/R injury, MMP levels in the TMZ-treated groups were similar to those of the IRC groups, which were all significantly greater than those of the sham groups (Figures 7(a)–7(c)). TIMP levels in the TMZ-treated groups became also comparable to those of the IRC groups, which were significantly greater than those of the sham groups (Figures 7(a), 7(d), and 7(e)).

## 4. Discussion

In the current study investigating the impact of post I/R treatment with TMZ on AKI and its progression to renal fibrosis against renal I/R injury in a rat model, we observed significant attenuation of renal injury by TMZ at short (24 h) as well as intermediate (5 d) period after renal I/R injury. Contrary to its early effects, however, post I/R treatment with TMZ, either given once or daily for 8 w, could not further suppress the progression to renal fibrosis and expressions of the related signaling pathways compared to those of the IRC group.

AKI is a major risk factor increasing morbidity and mortality in hospitalized patients. Notably, renal deterioration is gradually continued even after complete clinical recovery from AKI [4, 25]. Despite several favorable experimental results regarding the attenuation of renal I/R injury [24, 26, 27], the impracticability of providing treatments from 1 day to 1 month before the renal I/R injury stands opposed to the clinical translation of these results. Moreover, although attenuating the early injury is undoubtedly of great clinical importance, activated signals relevant to the immediate protective effects could promote injury or impede repair in the later stages of AKI [28, 29]. In that context, the finding that recombinant human erythropoietin delivered after reperfusion improved renal function and structure in the acute phase while it contributed to the development of CKD is worth noting [8].

TMZ is a commonly used antianginal drug with well-known cardioprotective effect and hemodynamic stability [30, 31]. Experimentally, TMZ also provided protection against renal I/R injury through its antioxidative, anti-inflammatory, and antiapoptotic effects [15, 32]. Of particular interest, TMZ inhibited delayed renal fibrosis through upregulation of HIF-1*α* in pigs after I/R injury [6]. Based on the theoretical advantages of TMZ and the clinical practicability of applying treatment after the onset of AKI, we investigated the effects of a single and repeated post I/R treatment of TMZ on AKI and its progression to renal fibrosis after renal I/R injury, while no comprehensive evidence exists in that regard.

In the current study, post I/R treatment with TMZ conferred renoprotective effects at 24 h and 5 d after I/R injury. Post I/R treatment with TMZ significantly mitigated apoptotic cell death and necrosis that were associated with inhibition of apoptotic pathways, enhanced expression of HIF-1*α*, and attenuated reduction in the VEGF level. TMZ treatment also resulted in upregulation of TIMPs and downregulation of MMPs. These results were consistent with those of previous studies that demonstrated the organ-protective effects of TMZ administered after ischemic insult on the heart, retina, and testis [33–35], mainly ascribed to its antioxidative property.

Contrary to the protective effects observed in the short- and intermediate-term, however, TMZ could not prevent renal fibrosis at 8 w after renal I/R injury in the present study. At 8 w, all protein expressions including VEGF, MMPs, and TIMPs as well as Bcl-2 and Bax were enhanced compared to those in the sham group. TMZ-induced early modulation of these signal protein expressions disappeared, resulting in comparable expression levels of all measured signal proteins among the IRC and TMZ-treated groups. These results are discordant with the results of previous reports showing that TMZ could reduce fibrosis development after I/R injury in pigs [6] and attenuate interstitial fibrosis in cyclosporine A-induced nephrotoxicity in rats [36].

A complex interplay of multiple mechanisms contributes to I/R-induced organ damage. In the hypoxic kidney, HIF-1*α* is expressed predominantly in tubular epithelial cells and works as a master regulator of hypoxic stress [7, 37]. In the current study, renal I/R injury elicited significant increase in the HIF-1*α* level, which was further increased by post I/R treatment with TMZ both at 24 h and 5 d after renal I/R injury. Despite the increased HIF-1*α* level, VEGF activities were significantly lower in the IRC group compared to that in the sham group, while its activities were significantly increased with TMZ treatment at 24 h and 5 d after renal I/R injury. In line with this, VEGF expression in the proximal tubules was reported to be lost early after AKI without treatment [38]. VEGF, regulated by HIF-1*α*, is involved in the regulation of the expression of many genes that help to reduce renal injury after I/R injury [4]. Thus, increased expression of HIF-1*α* accompanied by enhanced expression of VEGF with TMZ might contribute to the reduction of early renal injury after I/R in the present study. Of note, at 8 w after I/R injury, VEGF expressions were significantly greater in both the IRC and TMZ-treated groups than those of the sham groups, while HIF-1*α* could no more be detected in all groups. These findings indicate a delayed increase in VEGF independent of HIF-1*α*. Dysangiogenic VEGF, produced by intercellular proteolysis independent of HIF-1*α*, has been shown to elicit loss of endothelial integrity [39] and loss of nursing function of pericytes that stabilize capillaries [40]. Therefore, activated protective signals needed for the early phase but not removed in a timely manner may contribute to the continued production of profibrotic factors and the progression of renal fibrosis [8].

In the present study, TMZ attenuated expressions of MMPs and enhanced expressions of TIMPs at 5 d after I/R injury. Early after renal I/R injury, the influx of inflammatory mediators together with the degradation of necrotic cells induce upregulation of degradative enzymes in the renal interstitium [41–43]. Among the degradative enzymes, MMP-2 is particularly important. It has a high activity against collagen IV or basement membrane [44] and causes vascular endothelial damage [45]. Indeed, inhibition of MMPs has been shown to reduce I/R-induced AKI [41]. In that context, attenuated activation of MMPs with TMZ might have conveyed beneficial influence in mitigating the renal damage at 5 d after renal I/R injury in this study. After renal I/R injury, TIMPs, inhibitors of MMPs, are activated as well to counteract extracellular matrix degradation by MMPs. Therefore, the augmented activation of TIMPs at 5 d after renal I/R injury in the current study might have added favorable effects by suppressing the activities of MMPs in the TMZ-treated groups. On the other hand, expression levels of MMPs and TIMPs all remained greater in the IRC and the TMZ-treated groups than those of the sham groups at 8 w after renal I/R injury. Similar findings were observed in the previous study that assessed changes in the levels of MMPs and TIMPs for 24 w after renal I/R injury [46]. In that study, after an initial phase of increased extracellular matrix turnover following I/R injury, the balance subsequently turned towards the development of fibrosis, which was suggested to be the result of decreased extracellular matrix degradation by continuous activation of TIMPs [46]. Thus, our findings indicate that the offset of the balance between MMPs and TIMPs resulting in persistent enhancement of both molecules may have acted towards the development of interstitial fibrosis.

Contrary to our expectations, although a single dose was sufficient to exert early- and intermediate-term renoprotection, daily TMZ treatment did not exert additional effect over a single dose in the current study. Yet, considering that the elimination half-life of TMZ is approximately 6 h [47], employing an administration protocol of TMZ (2 to 3 times/day) yielding a steady state of the drug may have resulted in a different long-term outcome, which is a clear limitation of this study.

## 5. Conclusions

In conclusion, post I/R treatment with TMZ might allow ischemic kidneys to regain renal function and structure more rapidly compared to nontreated kidneys but not enough to resolute renal fibrosis in long-term aspect. Despite that, the early and intermediate renoprotective effects of TMZ treatment after I/R injury as affirmed by upregulation of HIF-1*α*, VEGF, and the inhibition of apoptotic pathways related to Bax deserve a clinical attention since the degree of recovery after AKI would significantly be associated with chronic renal function [48].

## Figures and Tables

**Table 1:** Parameters for semiquantitative assessment of histopathology.

	Cell degradation	Necrosis	Neutrophil infiltration
Grade 1	No change from normal	Nil	Nil
Grade 2	<5% of total field	Some single-cell necrosis	1–3 cells/field
Grade 3	5–30% of total field	Dispersed focal necrotic tubules	4–6 cells/field
Grade 4	>30% of total field	Confluent necrosis in most tubules	Heavy infiltration

**Table 2:** Renal histopathology assessed with PAS staining.

	Group	Cell degradation	Necrosis	Neutrophil infiltration
24 h	Sham	*Grade 1*	*Grade 1*	*Grade 1*
	IRC	2.6 ± 0.8^∗^	3.6 ± 0.5^∗^	1.3 ± 0.5
	TMZ^S^	1.7 ± 0.8^∗^^,#^	1.9 ± 0.7^∗^^,#^	1.1 ± 0.4
5 d	Sham	*Grade 1*	*Grade 1*	*Grade 1*
	IRC	3.1 ± 0.9^∗^	2.9 ± 0.9^∗^	1.9 ± 1.2
	TMZ^S^	2.1 ± 0.7	1.3 ± 0.5^#^	1.1 ± 0.4
	TMZ^D^	2.1 ± 0.7	1.6 ± 0.8^#^	*Grade 1*
8 w	Sham	*Grade 1*	*Grade 1*	*Grade 1*
	IRC	1.9 ± 0.4	1.1 ± 0.4	*Grade 1*
	TMZ^S^	1.4 ± 0.5	*Grade 1*	*Grade 1*
	TMZ^D^	1.1 ± 0.4	*Grade 1*	*Grade 1*

PAS = periodic acid-Schiff; TMZ = trimetazidine. Sham = rats not underwent ischemia-reperfusion; IRC = rats underwent ischemia (45 min)-reperfusion (24 h, 5 d, or 8 w); TMZ^S^ = rats treated with TMZ (3 mg/kg) upon reperfusion; TMZ^D^ = rats treated with TMZ (3 mg/kg) once daily for 5 d or 8 w starting upon reperfusion. ^∗^*P* < 0.05 versus sham, ^#^*P* < 0.05 versus IRC.

## Data Availability

Data will be made available on request.
